# Prevalence of nonalcoholic fatty liver disease and liver cirrhosis in Chinese adults with type 2 diabetes mellitus

**DOI:** 10.1111/1753-0407.13564

**Published:** 2024-04-25

**Authors:** Xinyu Han, Xin Zhang, Zhenqiu Liu, Hong Fan, Chengnan Guo, Haili Wang, Yu Gu, Tiejun Zhang

**Affiliations:** ^1^ Department of Epidemiology, School of Public Health Fudan University Shanghai China; ^2^ Key Laboratory of Public Health Safety (Fudan University) Ministry of Education Shanghai China; ^3^ State Key Laboratory of Genetic Engineering, Human Phenome Institute, School of Life Sciences Fudan University Shanghai China; ^4^ Fudan University Taizhou Institute of Health Sciences Taizhou China; ^5^ Shanghai Institute of Infectious Disease and Biosecurity, School of Public Health Fudan University Shanghai China; ^6^ Yiwu Research Institute Fudan University Yiwu China

**Keywords:** cirrhosis, HbA1c, nonalcoholic fatty liver disease, type 2 diabetes

## Abstract

**Background:**

Nonalcoholic fatty liver disease (NAFLD) and liver cirrhosis are significant clinical concerns, especially among individuals with type 2 diabetes mellitus (T2DM). However, in China, there is a paucity of reliable evidence detailing the characteristics of NAFLD and liver cirrhosis in T2DM. Furthermore, the relationship between blood glucose levels and NAFLD prevalence remains unclear.

**Methods:**

Data from the Shanghai Suburban Adult Cohort and Biobank were analyzed, including 6621 participants with T2DM. NAFLD was diagnosed by ultrasonography and liver cirrhosis was performed according to the health information systems. Logistic regression and restricted cubic spline analysis were used to explore the potential risk factors for NAFLD and liver cirrhosis.

**Results:**

The prevalence of NAFLD was 59.36%, and liver cirrhosis was 1.43% among T2DM patients. In these patients, factors like age, being female, marital status, and obesity significantly increased the risk of NAFLD. Specifically, obesity had a strong positive association with NAFLD (odds ratio [OR] = 4.70, 95% confidence interval [CI]: 4.13–5.34). The higher glycated hemoglobin (HbA1c) quartile was associated with a heightened NAFLD risk compared to the lowest quartile (all *p* < .001). The HbA1c‐NAFLD relationship displayed a linear that mimicked an inverted L‐shaped pattern. A significant positive association existed between HbA1c levels and NAFLD for HbA1c <8.00% (OR = 1.59, 95% CI: 1.44–1.75), but this was not observed for HbA1c >8.00% (OR = 1.03, 95% CI: 0.92–1.15).

**Conclusion:**

Systematic screening for NAFLD is essential in T2DM patients, especially with poor glucose control and obesity in female.

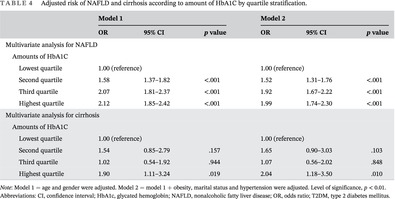

## Graphical abstract

Highlights

- A nonalcoholic fatty liver disease (NAFLD) prevalence of 59.36% and a liver cirrhosis prevalence of 1.43% was found among type 2 diabetes mellitus (T2DM) patients.
- Significant risk factors for NAFLD were identified, including age, gender, marital status, and obesity, with obesity showing a particularly strong positive association.
- The glycated hemoglobin (HbA1c)‐NAFLD relationship displayed a linear pattern that mimicked an inverted L‐shaped pattern. A significant positive association existed between HbA1c levels and NAFLD for HbA1c <8.00%, but this was not observed for HbA1c >8.00%.

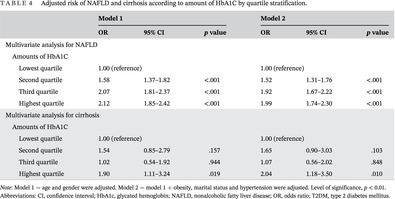

## 1 INTRODUCTION

Nonalcoholic fatty liver disease (NAFLD), a prevalent health concern affecting approximately one in four individuals worldwide,^1^, ^2^, ^3^ is a major cause of liver‐related morbidity and mortality, including being the fastest growing cause of hepatocellular carcinoma. Liver cirrhosis is a major health issue that afflicted more than 160 million people, and the highest incidence of liver cirrhosis was found in East Asia.^4^ Recently, NAFLD and liver cirrhosis have been found to be associated with obesity, insulin resistance, and metabolic syndrome.^5^, ^6^, ^7^, ^8^, ^9^

The global prevalence of type 2 diabetes mellitus (T2DM) is on the rise, affecting approximately 449 million people, and it is projected to rise to 702 million by 2045.^10^ Since 1980, diabetes prevalence in adults has either risen or stayed static in every country, leading to a nearly fourfold increase in global cases. The burden, in both prevalence and affected adults, has surged faster in low and middle‐income countries than in high‐income ones.^11^ China, in particular, has experienced a significant rise in T2DM prevalence since 1980, with a high proportion of cases undetected yet.^12^, ^13^ Previous epidemiological studies have consistently suggested the significance between T2DM and the risk of NAFLD.^14^, ^15^, ^16^, ^17^, ^18^, ^19^, ^20^ The increase of T2DM is so recent that its impact on the risk of liver diseases, if any, may not yet have fully emerged. Moreover, there is limited reliable evidence available in China on the associations of T2DM and blood glucose with risks of NAFLD. This knowledge gap, combined with uncertainties surrounding the prevalence of advanced fibrosis and cirrhosis in T2DM patients, has hindered the implementation of systematic screening guidelines as well.^20^

In the context of T2DM, glycated hemoglobin (HbA1c) control is a critical aspect of T2DM management, and HbA1c levels have been used as a key indicator of long‐term glucose control.^21^, ^22^ Maintaining lower HbA1c levels is associated with better T2DM management and reduced risk of complications.^23^, ^24^, ^25^ Meanwhile, HbA1c may contribute to the development of NAFLD as well, suggesting the potential value of glucose control in the administration of NAFLD. However, the relationship between HbA1c and NAFLD risk in T2DM patients remains to be fully elucidated. Previous studies found an association between random blood glucose levels and the risk of incident NAFLD, even in individuals without T2DM.^26^, ^27^ However, the relationship between HbA1c levels and the risk of NAFLD has not been consistent.

Furthermore, previous studies did not consistently demonstrate the gender differences on the prevalence of NAFLD or cirrhosis. Some earlier studies have indicated a higher NAFLD prevalence in males,^28^, ^29^, ^30^ whereas others have shown similar prevalence and incidence of NAFLD between genders or a higher prevalence in females.^31^, ^32^, ^33^ Therefore, large‐scale studies are highly warranted to better understand their association.

In this study, we aimed to investigate the prevalence of NAFLD and liver cirrhosis in the Chinese population with T2DM using data from a larger population‐based cohort and explore whether improved glucose control could potentially alleviate the occurrence of liver diseases.

## 2 METHODS

### 2.1 Study design

The present study was based on the Shanghai Suburban Adult Cohort and Biobank, which is a population‐based, large‐sized cohort study designed to provide a view of the health status of adults living in a suburban area.^34^ The baseline investigation started from June 2016 to April 2019. Participants aged between 20 and 74 years and living for at least 5 years in the district were recruited from Songjiang, Jiading, and Minghang district by stratified multistage random sampling. The cohort protocol was approved by the Ethics Committee on Medical Research at the School of Public Health, Fudan University (IRB#2016‐04‐0586).

**FIGURE 1:**
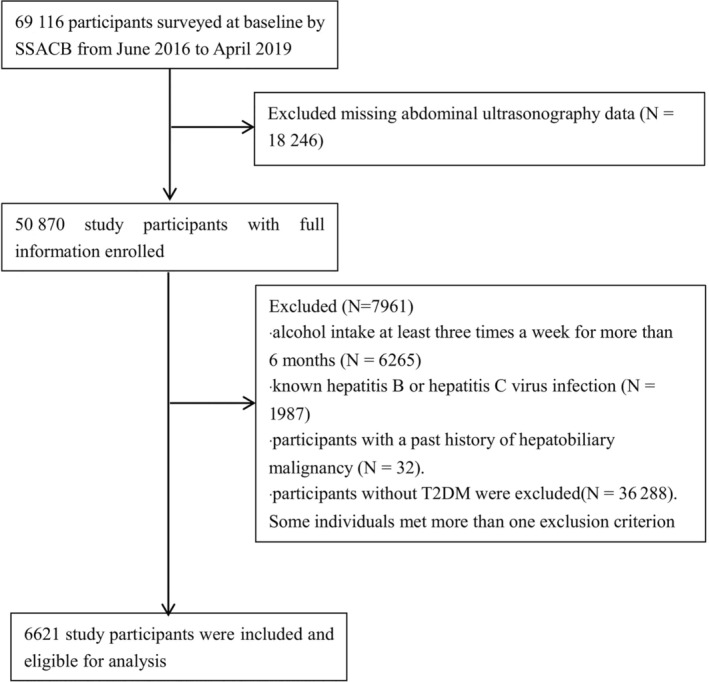
Selection process of the participants. SSACB, Shanghai Suburban Adult Cohort and Biobank; T2DM, type 2 diabetes mellitus.

Of the 69 116 participants at baseline, we excluded 26 207 participants for the following reasons: missing abdominal ultrasonography data (*N* = 18 246), alcohol intake at least three times a week for more than 6 months (*N* = 8265), or infected with hepatitis B or hepatitis C virus (*N* = 1987). We also excluded participants with a past history of hepatobiliary malignancy (*N* = 32) and participants without T2DM (*N* = 36 288). Because some individuals met more than one exclusion criterion, the total number of patients eligible for the study was 6621. The participant inclusion and exclusion criteria are depicted in Figure 1. All the participants signed written informed consent.

### 2.2 Study procedure and measurements

The survey collected data on demographic, socioeconomic, lifestyle, and disease history, and the physical examinations consisted of physiological measurements and laboratory testing. Trained staff conducted face‐to‐face interviews using a structured questionnaire to collect data on gender, age, education level, and marital and retirement status, as well as self‐reported chronic medical conditions such as hypertension, T2DM, hyperlipidemia, and coronary heart disease. The interviews were conducted on an Android tablet with audio recordings and paperless data entry to ensure accuracy and completeness of data. A random sample of 5% of the audio files was selected for quality checking. The use of statins was collected through the electronic medical record system, and the data were matched using a unique identification number (ID).

Anthropometric measurements were taken by clinicians using standardized methods to measure weight, height, waist circumference, hip circumference, and blood pressure. The blood samples were collected in the morning after at least 8 hours overnight fasting. The fasting plasma glucose was tested using an automated biochemistry analyzer with the hexokinase method, and HbA1c was measured by high‐pressure liquid chromatography using an automated glycated hemoglobin analyzer. To assess liver function, serum alanine aminotransferase and serum aspartate aminotransferase levels were measured by the Roche Cobas C702 using the International Federation of Clinical Chemistry and Laboratory Medicine and rate methods, respectively. Serum total protein and serum albumin were measured using the colorimetric method, serum creatinine was measured using the enzymatic method, and serum total bilirubin was measured using the diazo method. Serum lipids, including triglycerides, low‐density lipoprotein cholesterol (LDL‐C), high‐density lipoprotein cholesterol (HDL‐C), and total cholesterol, were measured using the colorimetric and enzymatic colorimetric methods on an automated biochemistry analyzer.

### 2.3 Diagnosis of diseases

T2DM was defined as fasting blood glucose >126 mg/dL or blood glucose >200 mg/dL or HbA1c ≥ 6.5% or with a self‐reported physician diagnosis of T2DM. NAFLD was defined according to widely accepted acoustic criteria: inconsistent liver and kidney echogenicity, the presence of increased liver echogenicity or liver brightening, poor penetration of the echogenicity into the deep liver, and vascular blurring, either singly or in combination. Liver cirrhosis was determined using a method combining *International Classification of Diseases* (ICD) diagnostic codes and electronic health records. Initially, we used ICD‐9 codes (571.5) and ICD‐10 codes (K74.0 and K74.1) to screen for potential cases of liver cirrhosis. Meanwhile, we conducted electronic health records reviews to further verify these cases and identify any missed cases. These involved reviewing clinical diagnostic records, imaging reports, and other relevant documentation for mentions of liver cirrhosis. Hypertension was defined as systolic blood pressure/diastolic blood pressure ≥140/90 mm Hg or with a previous diagnosis history.

### 2.4 Statistical analysis

The variables are expressed as mean ± SD, median (interquartile range [IQR]), or number and percentage (%). We used the chi‐square test to compare categorical variables and the Wilcoxon rank‐sum test or Kruskal–Wallis test to compare continuous variables between two or multiple groups. Additionally, to explore potential nonlinear relationships between certain predictor variables and the outcomes, we employed restricted cubic spline (RCS) analysis, which allows for more flexible modeling of continuous variables in logistic regression models.^35^ Specifically, we employed RCS to examine the potential nonlinear relationship between HbA1c levels and the odds of having NAFLD or liver cirrhosis. A multivariable logistic regression analysis was used to determine the association between the demographic factors (eg, gender, age, retirement status, education level, body mass index) and NAFLD/related liver cirrhosis. Corresponding odds ratios (ORs) and 95% confidence intervals (CIs) are reported as well. For all tests, statistical significance was defined as *p* < .01. We used Excel for data management and R software version 4.2.2 (R core, Vienna Austria) for statistical analyses.

## 3 RESULTS

### 3.1 Characteristics of the study population

The prevalence of NAFLD in the T2DM population was 59.4%, which was significantly higher than that in the whole population (38.0%) and in the non‐T2DM population (34.1%) (Table S1). A total of 6621 individuals with T2DM were included in the present study. The mean age of participants was 61.02 ± 8.15 years. Among them, 63.6% were female, 89.7% were married, and 18.0% had a high school education. Additionally, 75.4% were retired, 18.1% were smokers, and 29.0% were tea drinkers. The mean body mass index (BMI) was 25.58 ± 4.13 kg/m^2^, and obesity (BMI ≥28 kg/m^2^) was observed in 30.2% of the participants. The mean waist circumference was 85.98 ± 10.03 cm, and the mean hip circumference was 95.57 ± 15.19 cm. Regarding medical conditions, 53.1% had hypertension, 22.9% had hyperlipidemia, and 8.1% had coronary heart disease. Sex‐specific differences in prevalence of NAFLD in T2DM patients can be found in Table S2.

In terms of biochemical profiles, the median HbA1c level was 7.18% (IQR 6.50–7.70%). The median total bilirubin, albumin, triglycerides, HDL, LDL, platelet count, creatinine, total cholesterol, and total protein levels were 11.75 mg/dL, 49.11 g/dL, 2.05 mg/dL, 1.30 mg/dL, 2.80 mg/dL, 203.99 × 10^9^/L, 69.22 mg/dL, 5.02 mg/dL, and 76.90 g/dL, respectively.

### 3.2 Characteristics of NAFLD in T2DM

Table 1 displays the characteristics of the study participants stratified by the presence of NAFLD. Among all the 6621 T2DM participants, 59.36% (3930/6621) were with NAFLD. Table 3 presents the logistic regression analysis results investigating potential risk predictors for NAFLD in patients with T2DM. Specifically, gender was found to be significantly associated with NAFLD risk, with females having a higher risk compared to males (OR = 1.43, 95% CI: 1.23–1.65). The retirement status, smoking, tea drinking, and high school education rate did not demonstrate significant associations with NAFLD risk (*p* > .05). Obesity exhibited a substantial positive association with NAFLD risk (OR = 4.70, 95% CI: 4.13–5.34), and the association remained after adjusting for age and gender. The use of statins was also found to be significantly associated with NAFLD risk, with a higher risk compared to those who did not (OR = 1.39, 95% CI: 1.21–1.61).

**TABLE 1:** Basic characteristics of participants by NAFLD statues (*N* = 6621).

Characteristics	Total	No NAFLD	NAFLD	*p* value
	*N* = 6621	*N* = 2691	*N* = 3930	
Demographic and clinical				
Age in years, mean (SD)	61.02 ± 8.15	61.51 ± 8.35	60.69 ± 8.00	<.001
Female, *n* (%)	4212 (63.61)	1605 (59.64)	2609 (66.39)	<.001
Married, *n* (%)	5938 (89.65)	2358 (87.63)	3580 (91.09)	<.001
High school education rate, *n* (%)	1189 (17.96)	505 (18.76)	684 (17.40)	.161
Retirement, *n* (%)	4990 (75.37)	2045 (75.99)	2945 (74.94)	.338
Smoking, *n* (%)	1198 (18.09)	532 (19.77)	666 (16.95)	.004
Tea drinking, *n* (%)	1918 (28.97)	794 (29.51)	1124 (28.60)	.44
BMI (kg/m^2^), mean (SD)	25.58 ± 4.13	23.84 ± 4.57	26.79 ± 3.29	<.001
Obesity, *n* (%)	2000 (30.21)	358 (13.30)	1642 (41.78)	<.001
WC (cm), mean (SD)	85.98 ± 10.03	81.72 ± 8.45	88.91 ± 9.98	<.001
HC (cm), mean (SD)	95.57 ± 15.20	93.13 ± 21.89	97.29 ± 7.05	<.001
Race, *n* (%)	6612 (99.86)	2688 (99.89)	3924 (99.85)	.747
Hypertension, *n* (%)	3517 (53.12)	1234 (45.86)	2283 (58.09)	<.001
Hyperlipidemia, *n* (%)	1515 (22.88)	455 (16.91)	1060 (26.97)	<.001
CHD, *n* (%)	539 (8.14)	211 (7.84)	328 (8.35)	.245
Use of statins, *n* (%)	977 (14.7)	338 (12.5)	639 (16.3)	<.001
Biochemical profile, median (IQR)				
HbA1c (%)	7.18 (6.50–7.70)	7.00 (6.20–7.40)	7.31 (6.50–7.80)	<.001
AST (U/L)	22.65 (17.00–25.00)	20.45 (16.00–23.00)	24.32 (17.00–26.00)	.002
ALT (U/L)	24.33 (14.00–18.00)	19.40 (13.00–22.00)	27.70 (16.00–32.00)	<.001
Total bilirubin (mg/dL)	11.75 (8.20–14.20)	11.83 (8.30–14.20)	11.69 (8.20–14.20)	.301
Albumin (g/dL)	49.11 (47.10–51.20)	48.96 (46.90–51.10)	49.22 (47.20–51.30)	.001
Triglycerides (mg/dL)	2.05 (1.17–2.38)	1.62 (0.97–1.85)	2.34 (2.39–2.69)	<.001
HDL (mg/dL)	1.30 (1.07–1.50)	1.39 (1.14–1.61)	1.24 (1.03–1.43)	<.001
LDL (mg/dL)	2.80 (2.21–3.34)	2.79 (2.23–3.28)	2.80 (2.19–3.37)	.602
Platelet count (10^9^/L)	203.99 (164.00–239.00)	197.02 (157–231)	208.74 (171–244)	<.001
Creatinine (mg/dL)	69.22 (57.00–78.00)	70.55 (58.00–79.00)	68.31 (56.00–77.00)	<.001
TCHOL	5.02 (4.33–5.64)	4.92 (4.23–5.49)	5.10 (4.40–5.72)	<.001
TP	76.90 (74.00–79.90)	76.35 (73.50–79.30)	77.27 (74.50–80.30)	<.001

*Note*: *t* test performed on continuous variables presented as mean (SD), Wilcoxon rank‐sum test performed on all other continuous variables. Chi‐square or Fisher's exact test as appropriate on all categorical variables. Level of significance, *p* < .01.

Abbreviations: ALT, alanine aminotransferase; AST, aspartate aminotransferase; BMI, body mass index; CHD, coronary heart disease; HbA1c, hemoglobin A1c; HC, hip circumference; HDL, high‐density lipoprotein; IQR, interquartile range; LDL, low‐density lipoprotein; NAFLD, nonalcoholic fatty liver disease; T2DM, type 2 diabetes mellitus; TCHOL, total cholesterol; TP, total protein; WC, waist circumference.

### 3.3 Characteristics of liver cirrhosis in T2DM

Table 2 presents the demographic and clinical characteristics between individuals with and without liver cirrhosis. The results showed a prevalence of 1.43% (95/6621) for liver cirrhosis in patients with T2DM. Results from the logistic regression analysis indicated that individuals with obesity had a 1.84 times higher risk of developing cirrhosis compared to nonobese patients (OR = 1.84, 95% CI 1.22–2.79). Retirement, smoking, tea drinking, and high school education rate did not show significant associations with cirrhosis risk in this study population (Table 3).

**TABLE 2:** Demographic and clinical characteristics by liver cirrhosis (*N* = 6621).

Characteristics	No liver cirrhosis	Liver cirrhosis	
	*N* = 6526	*N* = 95	*p* value
Demographic and clinical			
Age in years, mean (SD)	60.99 ± 8.17	63.33 ± 6.64	.005
Female, *n* (%)	4160 (63.75)	54 (56.84)	.164
Married, *n* (%)	5865 (89.87)	72 (75.79)	<.001
High school education rate, *n* (%)	1169 (17.91)	20 (21.05)	.420
Retirement, *n* (%)	4910 (75.24)	79 (83.16)	.092
Smoking, *n* (%)	1180 (18.08)	17 (17.90)	.962
Tea drinking, *n* (%)	1878 (28.78)	40 (42.11)	.006
BMI (kg/m^2^), mean (SD)	25.57 ± 4.13	26.49 ± 3.06	.031
Obesity, *n* (%)	1958 (30.00)	42 (44.21)	.005
WC (cm), mean (SD)	85.87 ± 9.23	93.11 ± 33.43	<.001
HC(cm), mean (SD)	95.54 ± 15.29	97.96 ± 6.56	.001
Race, *n* (%)	6516 (99.85)	95 (100.0)	1.00
Hypertension, *n* (%)	3463 (53.06)	53 (55.78)	.607
CHD, *n* (%)	526 (8.06)	13 (13.68)	.126
Biochemical profile, median (IQR)			
HbA1c (%)	6.80 (6.50–7.70)	6.90 (6.60–8.40)	.031
AST (U/L)	20.00 (17.00–25.00)	20.00 (16.00–30.00)	.558
ALT (U/L)	19.00 (14.00–28.00)	22.00 (16.00–32.00)	.193
Total bilirubin (mg/dL)	10.90 (8.20–14.20)	10.50 (8.30–14.70)	.746
Albumin (g/dL)	49.20 (47.10–51.20)	49.40 (47.20–51.50)	.001
Triglycerides (mg/dL)	1.65 (1.17–2.38)	1.69 (1.25–2.71)	.559
HDL (mg/dL)	1.27 (1.07–1.51)	1.15 (0.98–1.38)	.002
LDL (mg/dL)	2.78 (2.21–3.34)	2.74 (2.09–3.28)	.971
Platelet count (109/L)	200.00 (165.00–239.00)	212.00 (165.00–239.00)	.520
Creatinine (mg/dL)	66.00 (57.00–78.00)	66.00 (57.00–80.00)	.614
TCHOL	4.97 (4.34–5.64)	4.80 (4.28–5.67)	.626
TP	76.90 (74.00–79.90)	78.00 (74.80–80.60)	.193

*Note*: *t* test performed on continuous variables presented as mean (SD), Wilcoxon rank sum test performed on all other continuous variables. Chi‐square or Fisher's exact test as appropriate on all categorical variables. Level of significance, *p* < 0.01.

Abbreviations: ALT, alanine aminotransferase; AST, aspartate aminotransferase; BMI, body mass index; CHD, coronary heart disease; HbA1c, hemoglobin A1c; HC, hip circumference; HDL, high‐density lipoprotein; IQR, interquartile range; LDL, low‐density lipoprotein; T2DM, type 2 diabetes mellitus; TCHOL, total cholesterol; TP, total protein; WC, waist circumference.

**TABLE 3:** Factors associated with NAFLD and cirrhosis in T2DM patients (*n* = 6621).

Variables	Risk for NAFLD			Risk for cirrhosis		
	OR	95% CI	*p* value	OR	95% CI	*p* value
Obesity (yes/no)	4.70	4.13–5.34	<.001	1.84	1.22–2.79	.004
Retirement (yes/no)	1.02	0.88–1.20	.770	1.29	0.65–2.54	.471
Married (yes/no)	1.51	1.27–1.78	<.001	2.79	1.72–4.54	<.001
Smoking (yes/no)	1.01	0.85–1.20	.918	0.69	0.37–1.30	.25
Tea drinking (yes/no)	1.10	0.97–1.25	.156	1.80	1.12–2.88	.015
High school graduation (yes/no)	1.01	0.88–1.16	.877	1.27	0.75–2.13	.377
Use of statins	1.39	1.21–1.61	<.001	1.24	0.72–2.05	.415

*Note*: Age and gender were adjusted. Level of significance, *p* < .01.

Abbreviations: CI, confidence interval; NAFLD, nonalcoholic fatty liver disease; OR, odds ratio; T2DM, type 2 diabetes mellitus.

### 3.4 Association between HbA1c Levels and NAFLD

**FIGURE 2:**
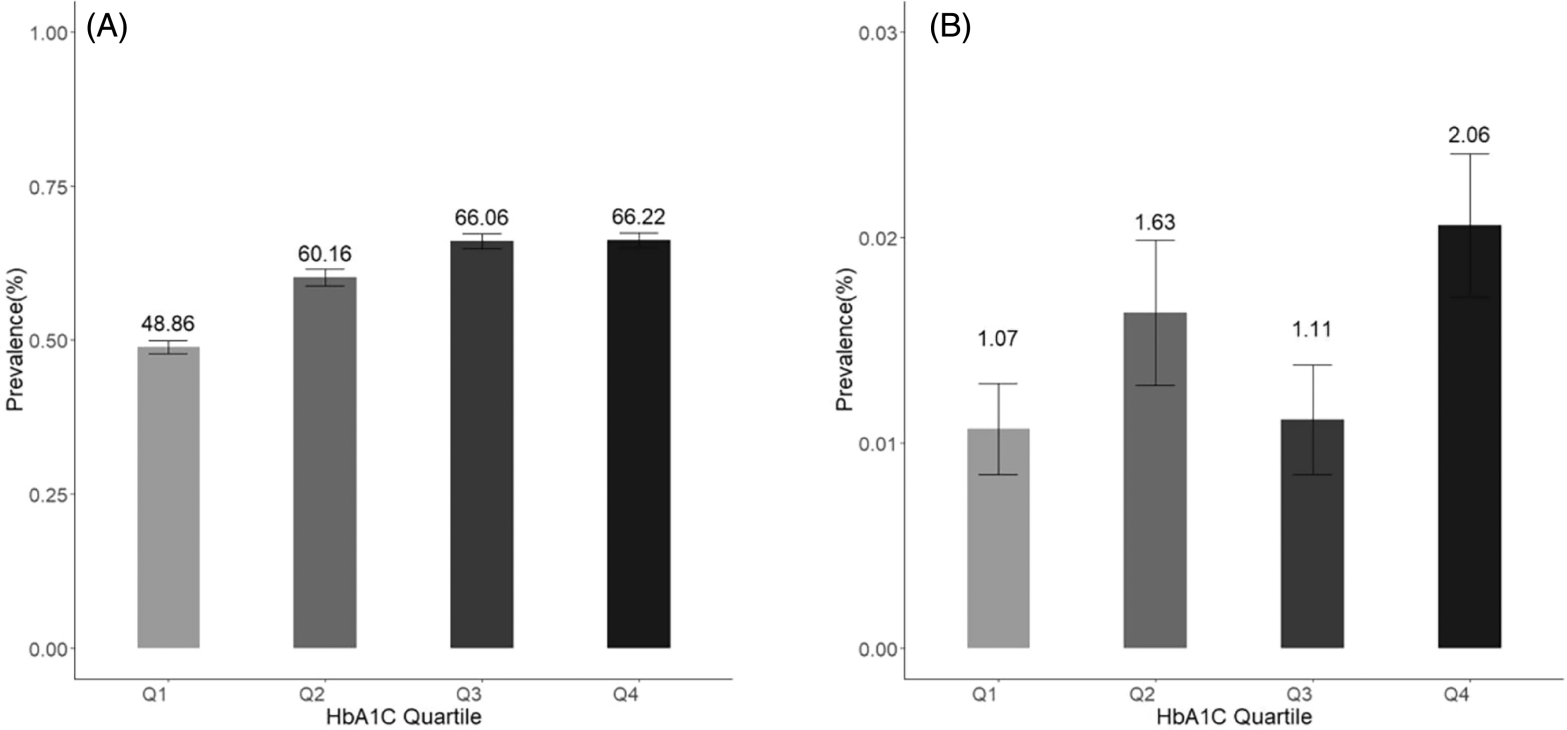
Prevalence of NAFLD (A), cirrhosis (B) in adults with T2DM according to amount of HbA1c by quartile stratification. HbA1c, glycated hemoglobin; NAFLD, nonalcoholic fatty liver disease; T2DM, type 2 diabetes mellitus.

Compared to the first quartile of HbA1c, the second, third, and highest quartiles were associated with a higher prevalence of NAFLD, with the OR (95% CI) increased by 1.58 (1.37–1.82), 2.07 (1.81–2.37), and 2.12 (1.85–2.42), respectively. (Table 4, Figure 2). The highest HbA1c quartile also showed a significant association with an increased risk of cirrhosis compared with the lowest quartile groups as the reference (OR = 1.90, 95% CI: 1.11–3.24). After adjusting for age, gender, obesity, marital status, and hypertension, consistent results were observed.

**TABLE 4:** Adjusted risk of NAFLD and cirrhosis according to amount of HbA1C by quartile stratification.

Variables	Model 1			Model 2		
	OR	95% CI	*p* value	OR	95% CI	*p* value
Multivariate analysis for NAFLD						
Amounts of HbA1C						
Lowest quartile	1.00 (reference)			1.00 (reference)		
Second quartile	1.58	1.37–1.82	<.001	1.52	1.31–1.76	<.001
Third quartile	2.07	1.81–2.37	<.001	1.92	1.67–2.22	<.001
Highest quartile	2.12	1.85–2.42	<.001	1.99	1.74–2.30	<.001
Multivariate analysis for cirrhosis						
Amounts of HbA1C						
Lowest quartile	1.00 (reference)			1.00 (reference)		
Second quartile	1.54	0.85–2.79	.157	1.65	0.90–3.03	.103
Third quartile	1.02	0.54–1.92	.944	1.07	0.56–2.02	.848
Highest quartile	1.90	1.11–3.24	.019	2.04	1.18–3.50	.010

*Note*: Model 1 = age and gender were adjusted. Model 2 = model 1 + obesity, marital status and hypertension were adjusted. Level of significance, *p* < 0.01.

Abbreviations: CI, confidence interval; HbA1c, glycated hemoglobin; NAFLD, nonalcoholic fatty liver disease; OR, odds ratio; T2DM, type 2 diabetes mellitus.

**FIGURE 3:**
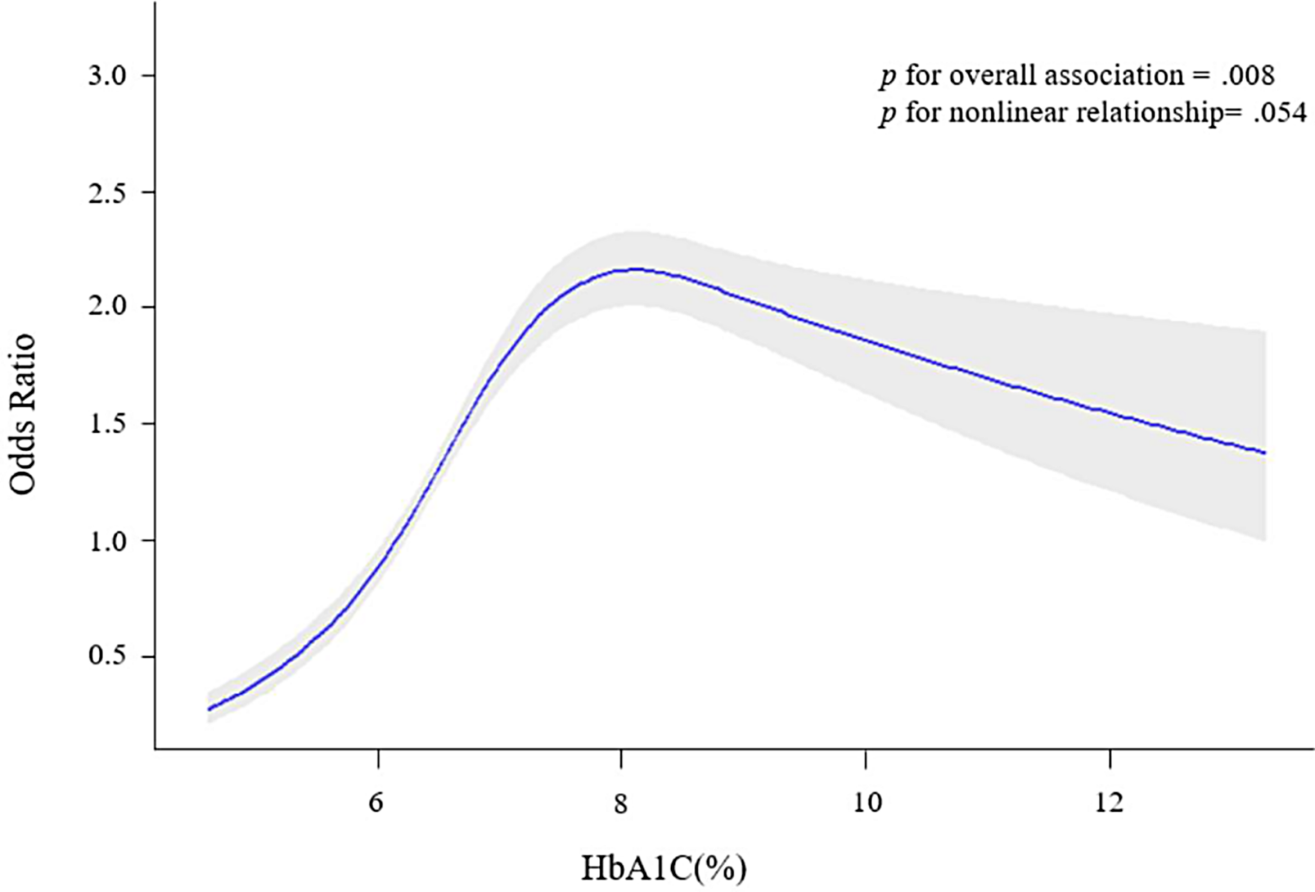
A Dose–response relationship of HbA1c and the risk of NAFLD. HbA1c, glycated hemoglobin; NAFLD, nonalcoholic fatty liver disease.

The dose–response plots derived from RCS methods demonstrated that the associations were nonlinear and mimicked an inverted L‐shaped pattern for the relationships between mean HbA1c and NAFLD (Figure 3). After adjusting for age, gender, obesity, marital status, and hypertension, the positive linear association between HbA1c levels and NAFLD was significant (OR = 1.59, 95% CI: 1.44–1.75) for HbA1c <8.00%, but not HbA1c >8.00% (OR = 1.03, 95% CI: 0.92–1.15) (Table 5).

**TABLE 5:** Association between HbA1c levels and NAFLD in adults with T2DM (linear relationship).

HbA1c levels	OR	95% CI	*p* value
HbA1c <8.0%	1.59	1.44–1.75	<.001
HbA1c >8.0%	1.03	0.92–1.15	.633

*Note*: ORs represent odds of more severe risk of NAFLD for every 1% increase in HbA1c. An ordinal logistic regression model was used and was adjusted for age, gender, obesity, marital status and hypertension. The choice of an HbA1c cutoff of 8.0% was data driven, based on dose–response plots (Figure 3).

Abbreviations: CI, confidence interval; HbA1c, glycated hemoglobin; NAFLD, nonalcoholic fatty liver disease; OR, odds ratio.

## 4 DISCUSSION

In the present study, we detected a higher prevalence (59.36%) of NAFLD and cirrhosis (1.43%) in the T2DM patients, predicting a potential risk for liver‐related morbidity and mortality among this population. The prevalence of NAFLD is consistent with previous reports.^14^ Previously, the presence of T2DM was found to be associated with more severe NAFLD, progressive disease, and liver‐related morbidity.^7^, ^8^, ^36^ Our study provides additional evidence on an Asian population based on a well‐established cohort. The findings of this study may contribute to the growing body of knowledge and underscore the importance of integrating liver health assessments into routine T2DM care.

HbA1c levels as a key indicator of glucose control are associated with better T2DM management and reduced complications risk.^37^ Generally, elevated HbA1c indicates persistent hyperglycemia, fostering NAFLD development,^38^ hepatic lipid accumulation, hepatic steatosis, and triggering inflammation, leading to liver cirrhosis. Findings of our study provide valuable insights into the relationship between HbA1c levels and the risks of NAFLD and liver cirrhosis in individuals with T2DM. Moreover, the dose–response plots indicated linear associations between HbA1c and NAFLD, highlight the importance of an optimal HbA1c threshold. The notable linear correlations indicate the importance of early intervention in blood glucose, particularly when HbA1c levels are below 8.00%. We observed a potential weakening of the association with HbA1c levels exceeding 8.0%, similar to another analogous study where the threshold was found to be 7%.^39^ The reasons for this disparity remain unclear. One possible explanation is that as the severity of fibrosis worsens, patients may lose the histological features of fatty liver, a phenomenon referred to as “burnt‐out NAFLD.” Patients with poor blood sugar control and severe fibrosis may have already lost the histological characteristics of fatty liver, leading to a nonlinear relationship with HbA1c. Another potential explanation is that with increasing HbA1c, there is a higher rate of antidiabetic medication usage. This is particularly relevant to NAFLD, as various antidiabetic medications are associated with a reduction in NAFLD.^40^ Our finding suggests the role of glycemic control in mitigating complications beyond the classic T2DM risk factors.

Interestingly, we found that women with T2DM bore a higher risk of NAFLD compared to men. This finding is consistent with a previous study conducted in 6648 Korean adults, which showed that women with fasting blood glucose ≥110 mg/dL or treated with glucose‐lowering medication had a higher risk of NAFLD than men.^29^ The reason for the increased risk of T2DM in women is unclear. However, studies have shown that greater lipid accumulation in visceral and liver tissues during glucose tolerance deterioration owing to the exhaustion of women's capacity to store fat in the subcutaneous depot may allow reaching the same visceral fat deposition required in men to develop NAFLD.^41^ Thus, lipid accumulation in the liver may facilitate both hepatic insulin resistance and hepatic inflammation, two key features of NAFLD.^42^, ^43^ These results suggest that gender differences in adiposity and other metabolic risk factors associated with worsening of glucose homeostasis likely contribute to gender differences in the driving force leading to NAFLD.

Research had found that tea is independently associated with NAFLD. Despite the diverse range of herbs included in herbal tea, such as chamomile and *Calicotome villosa*, and variations in preparation methods, a study by Carlsen et al^44^ highlighted that “herbal plants” rank among the most antioxidant‐rich food items and supplements globally, contributing significantly to antioxidant capacity as assessed by the ferric‐reducing ability of plasma. We hypothesize that the antioxidant capacity of herbal tea surpasses that of other teas, potentially exerting a more pronounced effect on liver health. Nevertheless, further studies are necessary to validate this observation. Additionally, animal experiments suggest that tea polyphenols may mitigate fibrosis, oxidative stress, and inflammation by targeting key inflammatory transcription factors and inhibiting hepatic stellate cell activation.^45^

Of note, our results also indicated that obesity is a crucial factor that increases the risk of NAFLD, especially in older adults with T2DM, highlighting the need for screening in this population. The strong link between obesity and NAFLD is consistent with previous report, indicating that excess adiposity contributes to the development and progression of hepatic steatosis. The accumulation of fat in the liver not only exacerbates insulin resistance but also triggers a cascade of inflammatory processes, leading to the more severe form of NAFLD, nonalcoholic steatohepatitis (NASH), and potentially progressing to liver cirrhosis. Previously, glucagon‐like peptide‐1 agonists have demonstrated effectiveness in treating T2DM and obesity and are showing promise in treating NASH,^46^ suggesting that systematic screening may identify potential therapeutic candidates for reducing metabolic and hepatic risk.

Despite the valuable insights generated by this study, limitations should be acknowledged as well. First, assessment of NAFLD was performed by liver ultrasound rather than the gold standard liver biopsy.^47^ However, liver biopsy is not feasible in large‐scale studies of NAFLD, whereas ultrasound provides a noninvasive, albeit less sensitive, widely available, and cheaper method to detect hepatic steatosis in clinical practice and research settings, as recommended by European Association for the Study of the Liver–European Association for the Study of Diabetes–European Association for the Study of Obesity Clinical Practice Guidelines. Second, we did not routinely test for autoantibodies. Nevertheless, considering that NAFLD typically arises in the context of insulin resistance, individuals with hyperglycemia and NAFLD are more likely to exhibit T2D physiology, even if they concurrently have type 1 diabetes mellitus (T1DM) or other uncommon forms of diabetes. Therefore, the potential misclassification of T2DM or inclusion of T1DM is unlikely to significantly affect the interpretation of our findings. Third, the single‐center design of our study may limit the generalizability of our findings to other populations. Further studies involving larger and more diverse cohorts from multiple centers are warranted to validate our results and enhance the external validity. It is also important to consider the potential bias stemming from the exclusion of individuals who did not undergo ultrasound examinations. To evaluate this, we compared demographic characteristics, namely age and gender, between individuals with and without ultrasound data. The absence of significant differences in these parameters supports the notion that excluding individuals without ultrasonography data did not introduce substantial bias regarding these demographic factors.

In conclusion, this study emphasizes the necessity of systematic screening among T2DM patients, especially those with poor blood glucose control and obese females. The results from our study provide substantial support for the perspective that T2DM is a dynamic state, and interventions to improve glycemic control can have a significant impact on reducing the risk of T2DM complications. From a public health perspective, the significance of addressing weight and glucose management should be emphasized to mitigate the risk of advanced liver disease. Future prospective studies are needed to further elucidate the optimal HbA1c target for reducing NAFLD risk and to unravel the underlying mechanisms linking HbA1c levels to NAFLD in a more comprehensive manner.

## AUTHOR CONTRIBUTIONS

All authors contributed to the study conception and design. Xinyu Han, Xin Zhang, and Yu Gu were responsible for data collection. Xinyu Han, Hong Fan, and Haili Wang were responsible for sorting and cleaning the data. Data analysis was performed by Zhenqiu Liu and Chengnan Guo. Xinyu Han is responsible for interpreting the results and drafting the article. Tiejun Zhang is responsible for revising it critically for important intellectual content and final approval.

## FUNDING INFORMATION

This study was supported by the Three‐Year Action Plan for Strengthening Public Health System in Shanghai (GWVI‐11.1‐23) and the Local High‐Level Discipline Construction Project of Shanghai.

## CONFLICT OF INTEREST STATEMENT

The authors declare that they have no conflicts of interest.

## Supporting information


**Table S1.** Characteristics of nonalcoholic fatty liver disease (NAFLD) in type 2 diabetes mellitus (T2DM) and non‐T2DM participants.
**Table S2.** Sex‐specific differences in prevalence of nonalcoholic fatty liver disease (NAFLD) in type 2 diabetes mellitus (T2DM) patients.
