# Supplementary material for: Prevalence of nonalcoholic fatty liver disease and liver cirrhosis in Chinese adults with type 2 diabetes mellitus
Source: J Diabetes. 2024 Apr 25;16(5):e13564. doi: 10.1111/1753-0407.13564 (PMC11045916; doi:10.1111/1753-0407.13564)
Supplement: Supplementary file 1 — Table S1. Characteristics of nonalcoholic fatty liver disease (NAFLD) in type 2 diabetes mellitus (T2DM) and non‐T2DM participants. Table S2. Sex‐specific differences in prevalence of nonalcoholic fatty liver disease (NAFLD) in type 2 diabetes mellitus (T2DM) patients. [file JDB-16-e13564-s001.docx]

**Supporting Information**

Table S1. Characteristics of NAFLD in T2DM and non-T2DM participants

|  | Non-T2DM | | | | | |  | T2DM | | | |
| --- | --- | --- | --- | --- | --- | --- | --- | --- | --- | --- | --- |
|  | Total N=36288 | No NAFLD N=23916 | NAFLD N=12372 | | p value |  | | Total N=6621 | No NAFLD N=2691 | NAFLD N=3930 | p value |
| **Demographic and clinical** |  |  | |  |  |  | |  |  |  |  |
| Age in years, mean (SD) | 55.13±11.94 | 54.64±12.48 | | 56.08±10.75 | ＜0.001 |  | | 61.02±8.15 | 61.51±8.35 | 60.69±8.00 | ＜0.001 |
| Female, n (%) | 24590(67.8) | 16275(68.1) | | 8315(67.2) | 0.105 |  | | 4212(63.6) | 1605(59.6) | 2609(66.4) | ＜0.001 |
| married, n (%) | 32959(90.80) | 21598(90.3) | | 11361(91.8) | ＜0.001 |  | | 5938(89.7) | 2358(87.6) | 3580(91.1) | ＜0.001 |
| High School Rate, n (%) | 9263(25.5) | 6353(26.6) | | 2910(23.5) | ＜0.001 |  | | 1189(18.0) | 505(18.8) | 684(17.4) | 0.161 |
| Retirement, n (%) | 21354(58.8) | 13649(57.1) | | 7705(62.3) | ＜0.001 |  | | 4990(75.4) | 2045(76.0) | 2945(74.9) | 0.338 |
| Smoking, n (%) | 5605(15.4) | 3732(15.6) | | 1873(15.1) | 0.251 |  | | 1198(18.1) | 532(19.8) | 666(16.9) | 0.004 |
| tea drinking, n (%) | 9117(25.1) | 5896(24.7) | | 3221(26.0) | 0.004 |  | | 1918(29.0) | 794(29.5) | 1124(28.6) | 0.440 |
| BMI (kg/m2), mean (SD) | 23.97±3.69 | 22.82±2.88 | | 26.18±4.06 | ＜0.001 |  | | 25.58±4.13 | 23.84±4.57 | 26.79±3.29 | ＜0.001 |
| Obesity (BMI ≥27kg/m2), n (%) | 6069(16.7) | 1754(7.3) | | 4315(34.9) | ＜0.001 |  | | 2000(30.2) | 358(13.3) | 1642(41.8) | ＜0.001 |
| WC(cm), mean (SD) | 80.76±10.05 | 77.87±9.67 | | 86.37±8.24 | ＜0.001 |  | | 85.98±10.03 | 81.72±8.45 | 88.91±9.98 | ＜0.001 |
| HC(cm), mean (SD) | 93.12±10.07 | 91.28±6.32 | | 96.69±14.18 | ＜0.001 |  | | 95.57±15.20 | 93.13±21.89 | 97.29±7.05 | ＜0.001 |
| Race, n (%) | 36220(99.8) | 23872(99.8) | | 12348(99.8) | 0.904 |  | | 6612(99.9) | 2688(99.9) | 3924(99.8) | 0.747 |
| Hypertension, n (%) | 10304(28.4) | 5552(23.2) | | 4752(38.4) | ＜0.001 |  | | 3517(53.1) | 1234(45.9) | 2283(58.1) | ＜0.001 |
| Hyperlipidemia, n (%) | 4027(11.1) | 1974(8.3) | | 2053(16.6) | ＜0.001 |  | | 1515(22.9) | 455(16.9) | 1060(27.0) | ＜0.001 |
| CHD, n (%) | 1598(4.4) | 980(4.1) | | 618(5.0) | ＜0.001 |  | | 539(8.1) | 211(7.8) | 328(8.3) | 0.245 |
| **Biochemical profile, median (IQR)** |  |  | |  |  |  | |  |  |  |  |
| HbA1c (%) | 5.58(5.30-5.90) | 5.54(5.30-5.80) | | 5.67(5.40-6.00) | ＜0.001 |  | | 7.18(6.5-7.7) | 7.00(6.20-7.40) | 7.31(6.50-7.80) | ＜0.001 |
| AST (U/L) | 22.18(17.00-24.00) | 21.58(17.00-24.00) | | 23.40(18.00-25.00) | 0.006 |  | | 22.65(17.00-25.00) | 20.45(16.00-23.00) | 24.32(17.00-26.00) | 0.002 |
| ALT (U/L) | 20.34(13.00-23.00) | 17.71(12.00-20.00) | | 25.44(15.00-29.00) | ＜0.001 |  | | 24.33(14.00-18.00) | 19.40(13.00-22.00) | 27.70(16.00-32.00) | ＜0.001 |
| Total bilirubin (mg/dl) | 11.66(8.10-14.10) | 11.70(8.20-14.10) | | 11.58(8.10-14.00) | 0.040 |  | | 11.75(8.20-14.20) | 11.83(8.30-14.20) | 11.69(8.20-14.20) | 0.301 |
| Albumin (g/dl) | 49.12(47.10-51.10) | 49.09(47.1-51.1) | | 49.18(47.20-51.20) | 0.009 |  | | 49.11(47.10-51.20) | 48.96(46.90-51.10) | 49.22(47.20-51.30) | 0.001 |
| Triglycerides (mg/dl) | 1.59(0.98-1.63) | 1.37(0.89-1.16) | | 2.02(1.26-2.38) | ＜0.001 |  | | 2.05(1.17-2.38) | 1.62(0.97-1.85) | 2.34(2.39-2.69) | ＜0.001 |
| HDL (mg/dl) | 1.41(1.17-1.63) | 1.47(1.23-1.66) | | 1.29(1.07-1.48) | ＜0.001 |  | | 1.30(1.07-1.50) | 1.39(1.14-1.61) | 1.24(1.03-1.43) | ＜0.001 |
| LDL (mg/dl) | 2.78(2.23-3.26) | 2.74(2.2-3.3) | | 2.86(2.30-3.38) | ＜0.001 |  | | 2.80(2.21-3.34) | 2.79(2.23-3.28) | 2.80(2.19-3.37) | 0.602 |
| Platelet count (109/L) | 204.65(166.00-239.00) | 201.08(163-235) | | 211.57(174-245) | ＜0.001 |  | | 203.99(164.00-239.00) | 197.02(157-231) | 208.74(171-244) | ＜0.001 |
| Creatinine (mg/dL) | 69.24(58.00-78.00) | 69.16(58.00-77.00) | | 69.42(58.00-78.00) | 0.249 |  | | 69.22(57.00-78.00) | 70.55(58.00-79.00) | 68.31(56.00-77.00) | ＜0.001 |
| TCHOL | 4.91(4.28-5.47) | 4.84(4.22-5.38) | | 5.06(4.43-5.16) | ＜0.001 |  | | 5.02(4.33-5.64) | 4.92(4.23-5.49) | 5.10(4.40-5.72) | ＜0.001 |
| TP | 76.45(73.60-79.40) | 76.24(73.40-79.20) | | 76.87(74.00-79.80) | ＜0.001 |  | | 76.90(74.00-79.90) | 76.35(73.50-79.30) | 77.27(74.50-80.30) | ＜0.001 |

*t* test performed on continuous variables presented as mean (SD), Wilcoxon rank sum test performed on all other continuous variables. Chi-square or Fisher’s exact test as appropriate on all categorical variables. Level of significance, *p* < 0.01. BMI, body mass index; WC, waist circumference; HC, hip circumference; HbA1c, hemoglobin A1c; ALT, alanine aminotransferase; AST, aspartate aminotransferase; HDL, high-density lipoprotein; LDL, low-density lipoprotein; TCHOL, total cholesterol; TP, total protein; NAFLD, nonalcoholic fatty liver disease; T2DM, type 2 diabetes mellitus; CHD, Coronary Heart Disease.

Table S2. Sex-specific differences in prevalence of NAFLD in T2DM patients

|  | Female | | |  | Male | | |
| --- | --- | --- | --- | --- | --- | --- | --- |
|  | No NAFLD N=1605 | NAFLD N=2609 | p value |  | No NAFLD N=1086 | NAFLD N=1321 | p value |
| **Demographic and clinical** |  |  |  |  |  |  |  |
| Age in years, mean (SD) | 61.68±7.75 | 61.20±7.30 | 0.044 |  | 61.25±9.14 | 59.67±9.14 | ＜0.001 |
| married, n (%) | 1370(85.4) | 2368(90.8) | ＜0.001 |  | 988(91.0) | 1212(91.7) | 0.512 |
| High School Rate, n (%) | 252(15.7) | 343(13.1) | 0.023 |  | 253(23.3) | 341(25.8) | 0.168 |
| Retirement, n (%) | 1354(84.4) | 2182(83.6) | 0.546 |  | 691(63.6) | 763(57.8) | 0.004 |
| Smoking, n (%) | 1(0.1) | 12(0.5) | 0.048 |  | 531(48.9) | 654(49.5) | 0.796 |
| tea drinking, n (%) | 202(12.6) | 368(14.1) | 0.161 |  | 592(54.5) | 756(57.2) | 0.181 |
| Obesity , n (%) | 208(13.0) | 1096(42.0) | ＜0.001 |  | 150(13.8) | 546(41.3) | ＜0.001 |
| WC(cm), mean (SD) | 80.22±8.31 | 87.41±8.67 | ＜0.001 |  | 83.92±8.13 | 91.86±11.59 | ＜0.001 |
| HC(cm), mean (SD) | 92.08±6.83 | 96.73±7.20 | ＜0.001 |  | 94.65±33.3 | 98.41±6.59 | ＜0.001 |
| Race, n (%) | 1603(99.9) | 2604(99.8) | 0.716 |  | 1086(99.9) | 1320(99.9) | 1.000 |
| Hypertension, n (%) | 754(47.0) | 1552(59.5) | ＜0.001 |  | 480(44.2) | 731(55.3) | ＜0.001 |
| Hyperlipidemia, n (%) | 286(17.8) | 685(26.3) | ＜0.001 |  | 169(15.6) | 375(28.4) | ＜0.001 |
| CHD, n (%) | 125(7.8) | 212(8.1) | 0.726 |  | 86(7.9) | 116(8.8) | 0.461 |
| **Biochemical profile, median (IQR)** |  |  |  |  |  |  |  |
| HbA1c (%) | 6.60(6.20-7.20) | 6.90(6.50-7.70) | ＜0.001 |  | 6.80(6.30-7.70) | 7.00(6.50-8.00) | ＜0.001 |
| AST (U/L) | 18.00(16.00-21.00) | 21.00(17.00-27.00) | ＜0.001 |  | 19.00(17.00-24.75) | 21.00(17.00-25.00) | 0.155 |
| ALT (U/L) | 15.00(12.00-20.00) | 21.00(15.00-31.00) | ＜0.001 |  | 18.00(14.00-24.00) | 24.00(17.00-36.00) | ＜0.001 |
| Total bilirubin (mg/dl) | 10.20(7.90-13.00) | 10.40(7.90-13.50) | 0.277 |  | 12.10(9.00-15.50) | 11.80(8.90-15.10) | 0.155 |
| Albumin (g/dl) | 49.00(47.00-51.10) | 49.30(47.10-51.20) | 0.044 |  | 49.10(46.90-51.20) | 49.40(47.50-51.60) | 0.002 |
| Triglycerides (mg/dl) | 1.37(1.02-1.97) | 1.91(1.41-2.67) | ＜0.001 |  | 1.24(0.90-1.74) | 1.88(1.37-2.75) | ＜0.001 |
| HDL (mg/dl) | 1.46(1.22-1.66) | 1.28(1.12-1.51) | ＜0.001 |  | 1.24(1.05-1.43) | 1.09(0.93-1.28) | ＜0.001 |
| LDL (mg/dl) | 2.88(2.37-3.42) | 2.88(2.27-3.46) | 0.106 |  | 2.54(2.09-3.08) | 2.65(2.07-3.19) | 0.062 |
| Platelet count (109/L) | 200.00(165.00-238.00) | 211.00(174.00-251.00) | ＜0.001 |  | 182.00(149.00-222.00) | 195.00(163.00-233.00) | ＜0.001 |
| Creatinine (mg/dL) | 61.00(54.00-68.00) | 60.00(52.00-68.00) | 0.041 |  | 78.00(70.00-87.00) | 79.00(70.00-89.00) | 0.605 |
| TCHOL | 5.11(4.49-5.75) | 5.17(4.55-5.81) | 0.045 |  | 4.48(3.97-5.07) | 4.77(4.19-5.43) | ＜0.001 |
| TP | 76.70(73.90-79.90) | 77.90(74.90-80.80) | ＜0.001 |  | 75.70(72.90-78.59) | 76.40(73.52-79.20) | ＜0.001 |

*t* test performed on continuous variables presented as mean (SD), Wilcoxon rank sum test performed on all other continuous variables. Chi-square or Fisher’s exact test as appropriate on all categorical variables. Level of significance, *p* < 0.01. BMI, body mass index; WC, waist circumference; HC, hip circumference; HbA1c, hemoglobin A1c; ALT, alanine aminotransferase; AST, aspartate aminotransferase; HDL, high-density lipoprotein; LDL, low-density lipoprotein; TCHOL, total cholesterol; TP, total protein; NAFLD, nonalcoholic fatty liver disease; T2DM, type 2 diabetes mellitus; CHD, Coronary Heart Disease.
